# Safety of Hepatitis B Vaccines (Monovalent or as Part of Combination) in Preterm Infants: A Systematic Review

**DOI:** 10.3390/vaccines12030261

**Published:** 2024-03-01

**Authors:** Qiao Wen Tee, Ramin Odisho, Elisha Purcell, Rachael Purcell, Jim Buttery, Claudia A. Nold-Petry, Marcel F. Nold, Atul Malhotra

**Affiliations:** 1 Department of Paediatrics, Monash University, 246 Clayton Road, Clayton, Melbourne, VIC 3168, Australiarodi0002@student.monash.edu (R.O.); elishapurcell003@gmail.com (E.P.); claudia.nold@hudson.org.au (C.A.N.-P.); 2 Infection Control and Epidemiology, Monash Health, Melbourne, VIC 3168, Australia; rachael.purcell@monashhealth.org; 3 Centre for Health Analytics, Melbourne Children’s Campus, Melbourne, VIC 3052, Australia; jim.buttery@mcri.edu.au; 4 Department of Paediatrics, University of Melbourne, Melbourne, VIC 3052, Australia; 5 The Ritchie Centre, Hudson Institute of Medical Research, Melbourne, VIC 3168, Australia; 6 Monash Newborn, Monash Children’s Hospital, Melbourne, VIC 3168, Australia

**Keywords:** adverse events, hepatitis B vaccine, preterm infants, neonate, timing

## Abstract

**Introduction**: The World Health Organization (WHO) recommends vaccination against hepatitis B as soon as possible following birth for all infants, regardless of prematurity. Hepatitis B vaccination at birth is clearly justified, represents a crucial step in the global control of perinatally acquired hepatitis B and there are no safety concerns in infants born at term. However, there is limited information on the safety of the hepatitis B vaccine in preterm infants, whose immune responses and morbidity risk differ from those in infants born at term. **Objectives**: The objectives of this paper are to systematically review the literature regarding the safety and risk of adverse events following immunisation (AEFIs) associated with the administration of the hepatitis B vaccine (monovalent or as part of a combination vaccine) to preterm infants. **Methods**: We performed a search for relevant papers published between 1 January 2002 and 30 March 2023 in the Ovid MEDLINE, Ovid Embase, Cochrane Central Register of Controlled Trials and CINAHL Plus databases. Two authors independently reviewed and analysed each article to include in the systematic review. Narrative synthesis is presented. **Results**: Twenty-one relevant papers were identified and included in this systematic review. The vast majority of data pertained to multi-antigen (combination) vaccine preparations and vaccination episodes from 6 weeks of age onwards. We found no publications investigating the timing of the birth dose of the hepatitis B vaccine, and AEFI reporting was exclusively short-term (hours to days following administration). There was substantial variability in the reported rate of AEFIs between studies, ranging from 0% to 96%. Regardless of frequency, AEFIs were mostly minor and included injection site reactions, temperature instability and self-limiting cardiorespiratory events. Six studies reported serious adverse events (SAEs) such as the requirement for escalation of respiratory support. However, these occurred predominantly in high-risk infant populations and were rare (~1%). Using the Grading of Recommendations, Assessment, Development, and Evaluations (GRADE) approach, the certainty of evidence was assessed as very low. **Conclusions**: Despite substantial variability between the relatively small number of published studies in terms of cohort selection, definitions, vaccine preparations and reporting, hepatitis B-containing vaccines (mostly as combination vaccines) appear to be relatively well tolerated in preterm infants from 6 weeks of age. Research focusing on the safety of hepatitis B vaccine in preterm infants specifically within 7 days of birth is lacking, particularly regarding long-term morbidity risk. Further research in this area is required.

## 1. Introduction

Hepatitis B is an important, vaccine-preventable viral infection affecting 257 million people living with chronic hepatitis B, and is responsible for 887,000 deaths a year [1]. This enveloped, partially double-stranded DNA virus can be transmitted in a variety of ways, including through contact with infected blood or bodily fluids [2]. The majority of hepatitis B transmission occurs vertically from a hepatitis B-positive mother, wherein the virus is transmitted from mother to child during pregnancy, delivery or breastfeeding via crackled nipples [3]. Infection in the newborn or during early childhood leads to chronic hepatitis in 95% of infections, whereas infection acquired during adulthood is less likely to lead to chronic disease, with less than 5% of patients in this category [4]. Childhood immunisation including a birth dose of the hepatitis B vaccine is included as two of the five core interventions in the WHO global control plan. As vertical transmission of hepatitis B can be prevented through appropriate vaccination of the infant at birth, timely vaccination is important.

The World Health Organization (WHO) recommends that all infants, regardless of prematurity, be vaccinated against hepatitis B as soon as possible after birth, preferably within 24 h, and an additional 2 or 3 doses of the vaccine are scheduled at least 4 weeks apart. The first dose of this vaccine is often given as a single-antigen product, whereas subsequent doses are most commonly given in combination, for example, with diphtheria, tetanus, pertussis (DTP), polio and *Haemophilus influenzae* type b (Hib) [5]. Notably, from a global perspective, adherence to vaccination as per national immunisation programs (NIPs) decreases with each scheduled dose. For example, the birth dose of the vaccine is usually given; however, subsequent vaccination rates decrease [6].

The preterm infant’s immune system is physiologically adjusted to life in the sterile intrauterine environment, and adaptation to dealing with extrauterine challenges takes time [7,8]. Moreover, depending on the degree of prematurity and the associated degree of maladaptation to the extrauterine life of other organ systems, preterm infants require substantial and prolonged medical support. Together, the unfit organs attempting to sustain life and adverse effects of such medical support commonly trigger local and systemic inflammation, which in turn contributes to multiple diseases of prematurity that, for instance, affect the brain, lung, heart, gut and eyes. It is important to consider how vaccination might interact with these circumstances [9]. Notably, the situation is different in term infants, who are more likely to be immunised on time (as compared to deferred vaccines in preterm infants), and who tend to tolerate the vaccines better.

We recently published that type 2-polarised inflammation (i.e., the dominance of a particular pathway of immune activation) is a major driver of bronchopulmonary dysplasia (BPD) and BPD-associated pulmonary hypertension (BPD-PH), both cardiopulmonary diseases that affect preterm infants [8]. Notably, we observed a strongly positive correlation between the signature cytokine of type 2 polarisation, IL-4, in T cells and hepatitis B vaccination when administered early, i.e., within the first few hours of life (e.g., odds ratio (OR) of 10.8 if hepatitis B vaccine was given within the first 24 h compared with no vaccination (95% confidence interval (CI) 2.9–4.0, *p* = 0.0004)) [8]. In contrast, vaccination at approximately day 7 of life was not associated with increased type 2 polarisation. Type 2-polarised inflammation was also strongly associated with BPD in the infants we studied, and we demonstrated the causality of this type of inflammation driving BPD in a murine model of disease (in which BPD was significantly prevented when type 2 inflammation was blocked) [8]. Therefore, these results raised an important question regarding the safety of early hepatitis B vaccination in preterm infants, especially those at risk of BPD.

In a previous systematic review [10], we noted that the timing of the first dose of the hepatitis B vaccine in preterm infants is highly variable around the world. Only 13% of the guidelines we identified and reviewed recommended a birth dose of the hepatitis B vaccine for all infants, regardless of prematurity. In 40% of the guidelines, the birth dose was only recommended for infants with a birth weight of more than 2000–2200 g; in 33% of the guidelines, there was no generalised birth dose recommended for all infants, and another 13% of guidelines had variable recommendations for this vaccine.

Hardly any research is published surrounding the safety of hepatitis B vaccination soon after birth in preterm babies. Adverse events following immunisation (AEFIs) are untoward events that occur following vaccination, irrespective of causality, while adverse effects are those where causality has been established. Based on our previous paper [10], it is clear that the timing of hepatitis B vaccination around the world is variable, and these circumstances call for a look at the literature for the safety of hepatitis B vaccination at different time points of administration. Accordingly, we aimed to assess the safety of the hepatitis B vaccine (as a monovalent or a combination vaccine) in infants. In doing so, we considered the AEFIs of the hepatitis B vaccine, specifically in association with the time point of administration in preterm infants at risk of BPD in addition to other long-term AEFIs of the vaccine.

## 2. Methods

This systematic review followed the Preferred Reporting Items for Systematic Reviews and Meta-analyses (PRISMA) [11].

### 2.1. Eligibility Criteria

Human studies of any study design investigating the safety of hepatitis B vaccination in preterm infants, defined as infants born less than 37 weeks of gestation, were included in this review. Studies of both term and preterm infants were included, provided the paper reported results of the preterm infant group. Studies investigating safety outcomes of the hepatitis B vaccination as a monovalent product or in combination with other vaccine products were eligible for this review. Relevant studies published after 1 January 2002 were included, even if participants were born prior to 1 January 2002. The exclusion criteria included protocols, literature reviews, conference abstracts, studies on animal models, studies not specific to preterm infants, studies investigating an intervention that did not include the hepatitis B vaccine, studies where the full text was not accessible in English and studies published prior to 1 January 2002.

### 2.2. Search Strategy

A search was initially performed broadly to identify articles related to the safety of vaccines given to preterm babies at any age. This search was subsequently narrowed to the safety of hepatitis B vaccines in preterm babies, and later further refined to the safety of hepatitis B vaccines in preterm babies given at or soon after birth. Relevant articles published between 1 January 2002 and 30 March 2023 were identified by searching the Ovid MEDLINE, Ovid Embase, Cochrane Central Register of Controlled Trials and CINAHL Plus databases. The search strategy is outlined in Supplementary Table S1.

### 2.3. Study Selection

The search results from all databases were imported into the Covidence systematic review software (https://www.covidence.org/). At least two independent authors performed title and abstract screening to determine whether each article was to be included in the review, based on the inclusion and exclusion criteria. Any discrepancies in reviewer decisions were resolved via discussion with a third reviewer. This process was then applied to full-text screening.

### 2.4. Data Collection

Relevant articles identified were extracted and analysed by two independent reviewers. Information extracted from studies included study population, study design, methods, vaccine used, results and conclusions. This information was further summarised, with the primary outcome being the safety effects of the hepatitis B vaccine and a narrative synthesis of data was conducted. Definitions and terminology of AEFI and serious adverse events (SAEs) associated with vaccine administration were reported in accordance with The Definition and Application of Terms for Vaccine Pharmacovigilance, a report written by the Council for International Organizations of Medical Sciences and World Health Organization [12].

### 2.5. Quality Assessment

The risk of bias in included studies was performed using the Risk of Bias In Non-randomised Studies of Interventions (ROBINS-I) [13,14]. For each study, two independent reviewers assessed the risk of seven types of bias. These were bias due to confounding, bias in the selection of participants into the study, bias in classification of interventions, bias due to deviation from intended interventions, bias due to missing data, bias in measurement of outcomes and bias in selection of the reported results. Discrepancies between reviewer judgements were resolved through discussion with a third reviewer. The overall quality of evidence of included studies was evaluated using the Grading of Recommendations Assessment, Development and Evaluation (GRADE) approach [15,16].

## 3. Results

### 3.1. Study Selection

**Figure 1:**
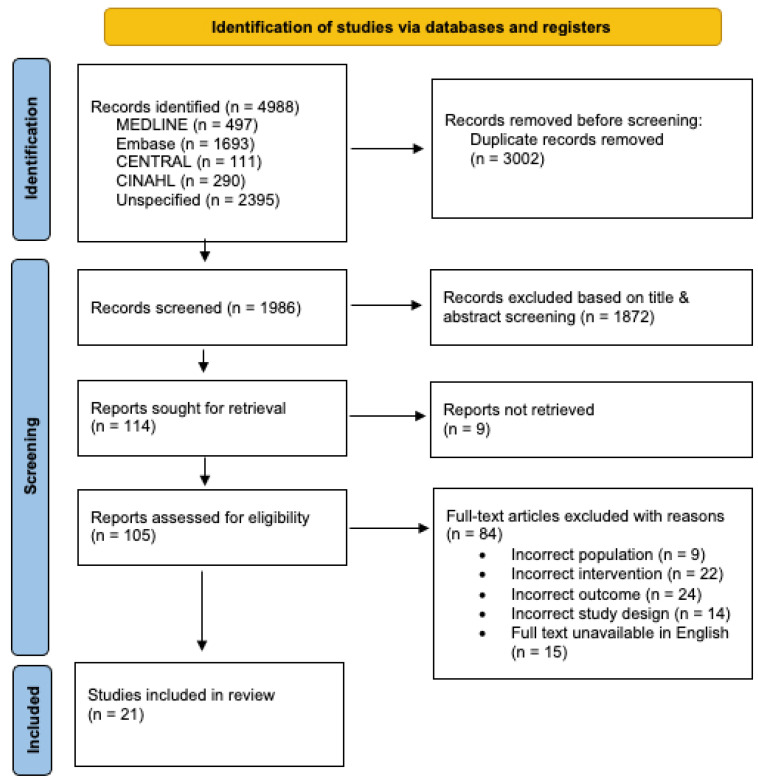
PRISMA flow chart summarising the study selection and screening process.

Our search strategy identified a total of 4988 studies, of which 2993 duplicate articles were automatically removed and 9 were manually removed, resulting in 1986 articles being screened. After title and abstract screening, 1872 studies were excluded based on the predefined selection criteria, and an additional 9 articles were unable to be retrieved in full text. There were 105 articles that underwent full-text screening, and 84 studies were excluded for the following reasons: wrong population (n = 9), wrong intervention (n = 22), wrong outcome, specifically no safety data (n = 42), wrong study design (n = 14) and full text unavailable in English (n = 15). In total, 21 studies were deemed eligible for inclusion in this review (Figure 1).

### 3.2. Study Characteristics

The characteristics of included studies are outlined in Table 1 and Table 2. Out of the total 21 studies identified, the most commonly used vaccine was Infanrix Hexa (DTPa-HBV-IPV/Hib, GSK Vaccines, Middlesex, UK) (n = 12). All but 3 studies reported on multi-antigen vaccines such as the hexavalent DTPa-IPV-HepB-Hib administered from 6 weeks of age or later as opposed to the single-target hepatitis B vaccine. All of the 21 studies we included reported exclusively on short-term AEFIs, typically covering the first 24 h to 1 week at most. Additionally, the vast majority of vaccination episodes did not use a single-antigen vaccine, but antigen combinations instead.

### 3.3. Safety of Hepatitis B Vaccine Administration in Preterm Infants

Seven studies reporting on a total number of 3655 infants showed a low rate of AEFIs (<10%, Table 1, Table 2 and Table 3). However, other studies reported somewhat different findings. For example, 9 other studies published AEFI rates greater than 10% in a total number of 1833 babies (Table 1 and Table 3), with one study observing non-cardiorespiratory AEFIs in up to 96% of vaccination episodes [32]. However, in each of these 15 studies, the reported AEFIs were mostly minor, including low-grade fever, local reactions such as rashes, local pain, erythema and swelling, and short-lived episodes of apnoea and bradycardia, which did not require escalation of support (total of 5488 babies; Table 1, Table 2 and Table 3).

In contrast, in certain patient groups, for example, infants who presented with apnoea following vaccination once [26] or infants who required in-hospital monitoring because of increased recurrence of cardiorespiratory events during the first immunisation [30], AEFIs were much more common (Table 1 and Table 3). In both studies reporting on such special cohorts, the recurrence of AEFIs was greater than 90%, and one infant in Clifford et al. developed a hypotonic hyporesponsive episode [26].

SAEs were reported to be associated with the vaccines containing the hepatitis B antigen in 6 studies [20,22,24,26,27,32] with a total number of 1051 infants (Table 1 and Table 3, SAEs highlighted in bold). Such SAEs comprised apnoea/bradycardia/desaturations (recurrent or at least requiring stimulation or intermittent positive pressure ventilation), which occurred in 8% to 18% of preterm infants following a multi-antigen vaccine containing hepatitis B. In 3 studies [22,26,27], escalation of respiratory support, including endotracheal intubation, use of CPAP or high flow, and admission to ICU was sometimes required. In [22], 9 of the 19 infants with a birth weight of less than 1500 g presenting with apnoea required endotracheal intubation and mechanical ventilation. Similarly, in a retrospective audit [27], of the 17 infants diagnosed with vaccine-related apnoea, 9 required re-admission to an intensive care unit for respiratory support with CPAP or high-flow nasal cannula. However, most of these SAEs were either reported in high-risk cohorts or in the studies that did identify them as relatively rare.

Besides our own study [8], which was not designed to address the question we are aiming to answer here, only two studies [35,36] reported on hepatitis B vaccination within 7 days of birth (Table 2). Neither of these studies made special mention of the birth dose, and AEFIs were either rare [36] or absent [35]. Hence, although 1666 infants who received a single-antigen hepatitis B vaccine at birth were included in studies we identified, there was no data to allow for any conclusions regarding the safety of hepatitis B vaccination at and around the time of birth in preterm infants.

Three studies explored the question of whether preterm birth predisposes infants to a higher risk of AEFIs in response to vaccinations ([19,32,34]; Table 1 and Table 3). In the 333 infants studied overall, this appeared not to be the case, as the occurrence of AEFIs was not significantly different between these groups. Similarly, this observation was also found in one study that reported on whether preterm birth predisposes infants to a higher risk of AEFIs when compared to term infants [32].

No studies reported on the specific impact of different timing strategies regarding hepatitis B vaccination in preterm infants upon the risk of BPD or on AEFIs following a first dose given in the first few hours of life. Moreover, we found no literature on long-term AEFIs, i.e., effects beyond hours to days. Further to this, there were no studies specifically focusing on the single-antigen hepatitis B vaccine on premature infants.

### 3.4. Quality Assessment

The risk of bias assessment of included studies is summarised in Supplementary Figure S1. Across the evaluated biases, most studies were judged as having low risk or unclear risk of bias due to a lack of information available. However, notably, 17 of a total of 21 studies were judged as having a moderate risk of bias in the measurement of outcomes. This judgement was based on the absence of investigator blinding across these studies. The GRADE system for rating the quality of evidence and strength of recommendation was used to assess the level of evidence of included studies. All studies began with an initial low quality of evidence (+2) due to their observational study designs. The certainty of evidence was downgraded (−1) due to the ‘serious’ inconsistency identified across the reported results of the 21 studies. Of note, the percentage of reported AEFIs varied from 0 to 90% across the 21 studies. In following the GRADE approach, the quality of evidence was not further downgraded as no serious risk of bias, indirectness, imprecision or publication bias was identified. The certainty of the evidence was not upgraded as no large effect, dose–response or all plausible confounding was identified. Thus, in following the GRADE approach, the overall quality of evidence included in this review was judged as very low.

## 4. Discussion

This systematic review was conducted to determine the safety of administering the hepatitis B vaccine (either monovalent or in combination vaccines) to preterm infants, especially soon after birth. This review was conducted on the background of our previous prospective immune profiling cohort study [8], raising the possibility that the birth dose of the single-antigen hepatitis B vaccine further increases the risk of BPD in preterm infants already at risk of developing this chronic cardiopulmonary disease. We did not find any of the literature investigating this specific question.

Birth dose hepatitis B vaccination policies for preterm infants are highly variable around the world [10]. Apart from the prevention of vertical transmission of hepatitis B—which of course is of critical importance, particularly in regions with high hepatitis B prevalence—there is no known added benefit of receiving a birth dose of the hepatitis B vaccine. For the development of immunogenicity in preterm infants, commencing hepatitis B vaccination at 6–8 weeks of life is sufficient, provided infants born <32 weeks of gestation or <2000 g birth weight receive an additional booster dose at 12 months of age [37]. Since our research has raised safety concerns in a specific patient group, i.e., preterm infants at risk for BPD, the question of the safety of the hepatitis B birth dose should be addressed as a matter of urgency.

The studies we identified were observational studies, with relatively small sample sizes, describing safety outcomes in the hours and days following hepatitis B vaccination. The hepatitis B antigen was almost always part of a multi-antigen (combination) vaccine preparation. Moreover, most studies did not include the birth dose, but commenced monitoring from the 6 week-time point onwards. We did not find any randomised studies or any research on long-term AEFIs. Therefore, it is difficult to determine the short-term safety of the hepatitis B vaccine at birth or in the first 6 weeks of life and is not possible to conclusively comment on its long-term safety. Thus, research in this field is required.

Overall, there were some consistent short-term temporal associations with vaccination administration, including minor local reactions and self-limiting cardiorespiratory events not requiring escalation of support after the 6-week to 2-month doses. Nonetheless, the published studies substantially diverge regarding the frequency of such minor AEFIs, ranging from 0 to 96%. The reported occurrence of SAEs, such as the requirement to escalate respiratory support, is similarly variable, with some studies reporting such SAEs and others not. These differences are likely due to cohort selection, duration of observation and variability in definitions and reporting. The importance of cohort selection in particular is demonstrated by studies such as Clifford et al. [26] and Bohnhorst et al. [30], which investigated higher-risk infants and consistently observed high AEFI rates. More definitive evidence in this regard would be useful, for example, to identify populations at higher risk of presenting with AEFIs, thus guiding monitoring requirements.

This review has several limitations. Almost all studies we reviewed reported on combination products including hepatitis B, typically with pertussis antigens, tetanus and diphtheria toxoids, Hib and polio. Therefore, it is nearly impossible to establish whether the AEFIs of these vaccines were related to the hepatitis B antigen/vaccine or other components of the preparation, possibly including adjuvants. Cardiorespiratory events similar to those identified in the studies included here have been described following pertussis-containing vaccines [38]. Furthermore, there are limitations regarding the implemented search strategy. In combining the search terms of ‘adverse event’, ‘safety’, ‘complication’, ‘surveillance’, ‘deterioration’ and ‘response’ with the Boolean operator ‘or’ studies that have used other terminology to describe safety outcomes may have been missed. Additionally, only studies where the full text was available in English were included in this study. In addressing such limitations, additional studies may have been eligible for this review and subsequently altered the overall conclusions made.

In summary, despite substantial variability between the relatively small number of published studies, hepatitis B-containing vaccines (mostly as a combination) appear to be relatively well tolerated in preterm infants from 6 weeks of age. There is minimal published information available on the safety of the birth dose of single-antigen (monovalent) hepatitis B vaccines in preterm infants, especially in terms of prematurity-related morbidities.

## 5. Conclusions

Due to the low number of published studies, low sample size, high variability in cohort selection, vaccine preparations and reporting, as well as very low certainty of evidence available, a conclusion is difficult to establish. Vaccination against hepatitis B at birth remains a critical component of global disease prevention, particularly in settings where maternal hepatitis B carriage is high and no safety concerns have been raised in infants born at term, who represent the vast majority of neonates worldwide. However, for the ~0.5% of neonates born prematurely who are at risk of BPD and for whom, therefore, hepatitis B vaccination at birth may carry an increased risk [8], there is a paucity of literature regarding the safety of this vaccine. Notably, guidelines in many regions with low hepatitis B prevalence and/or reliable antenatal screening programs and/or high vaccination rates in the first years of life already recommend not administering the birth dose to preterm infants (e.g., birth weight < 2000 g [10]). Given that the birth dose is not necessary for immunogenicity in preterm babies if a 12-month dose is used [31], the pragmatic approach already used in many regions appears sensible until reliable safety data are published.

## Figures and Tables

**Table 1:** Summary of safety outcomes of vaccines administered to preterm infants greater than 7 days post birth. Abbreviations used: AEFI, adverse event following immunisation; SAE, serious adverse event.

Title, Author, Year	Gestation at Birth	Time Vaccination Was Given Post Birth	Vaccine Administered	n	Outcomes Summary, Including Reported AEFIs Following Hepatitis B Immunisation
Adverse reactions to immunization with newer vaccines in the very preterm infant Ellison et al., 2005 [17]	≤30 weeks	2 months	Haemophilus b conjugate (Hib) (meningococcal protein conjugate) and hepatitis B (recombinant) vaccine (Comvax). Diphtheria, tetanus, acellular pertussis-containing vaccine (DTPa), hepatitis B (HepB) and inactivated poliovirus vaccine (IPV) (Infanrix).	48	AEFIs included low-grade fever in 16 (33%) infants after immunisation, 0% before immunisation. Side effects were monitored within 48 h of immunisation. No SAEs identified.
Apnoea and bradycardia in preterm infants following immunisation with pentavalent or hexavalent vaccines Schulzke et al., 2005 [18]	Mean 28 weeks	2 months	DTPa-IPV+Hib (Infanrix) or DTPa-HepB-IPV-Hib (Infanrix Hexa)	53	AEFIs included 13% (n = 7) who showed a transient recurrence of, or increase in, episodes of apnoea or bradycardia. No SAEs identified.
Response of preterm newborns to immunization with a hexavalent diphtheria-tetanus-acellular pertussis-hepatitis B virus-inactivated polio and Haemophilus influenzae type b vaccine: first experiences and solutions to a serious and sensitive issue Omenaca et al., 2005 [19]	Preterm infants born between 24 and 36 weeks and control group of full-term infants	2, 4 and 6 months	Hexavalent DTPa-HBV-IPV/Hib vaccine	92	AEFIs included some extremely preterm infants who experienced transient cardiorespiratory events within 72 h; 1 premature infant < 28 weeks had 2, and another infant of similar gestational age had 1 episode of apnoea within 24 h of immunisation, which resolved after stimulation. A total of 14.2% and 12.0% of doses were followed by fever in the preterm and full-term groups, respectively, but none >39.5 °C. The authors stated that vaccine was well tolerated overall. No SAEs identified.
Primary immunization of premature infants with gestational age <35 weeks: cardiorespiratory complications and C-reactive protein responses associated with administration of single and multiple separate vaccines simultaneously Pourcyrous et al., 2007 [20]	<35 weeks	2 months	DTaP, Hib. HBV, IPV and Pneumococcal conjugate vaccine (PCV7)	239	AEFIs included abnormal elevation of CRP occurred in 85% (multiple vaccines) and up to 70% (single vaccine). 39 infants (16%) had vaccine-associated cardiorespiratory events. Infants were monitored for 72 h for these events. 26 of these infants were initiated on O_2_ therapy or increase in FiO_2_. **SAEs** included 13 of the infants who had vaccine-associated cardiorespiratory events started on bag-mask ventilation, CPAP, mechanical ventilation or increase in ventilator settings.
Safety of DTaP-IPV-HIb-HBV hexavalent vaccine in very premature infants Faldella et al., 2007 [21]	<31 weeks	2 months	DTaP–IPV–HIb–HBV (Infanrix Hexa)	45	AEFIs included 5 infants (11%) who had apnoea and/or bradycardia and/or desaturation. No SAEs identified.
Apnoea and its possible relationship to immunization in ex-premature infants Cooper et al., 2008 [22]	Mean 30 weeks	6, 10 and 14 weeks	Bacille Calmette Guerin (BCG) and oral polio vaccine at birth. DTP-HepB Oral live, trivalent poliovirus types 1, 2 and 3.	23	AEFIs included 7 infants who developed apnoea within 72 h of vaccination possibly related to their vaccines, 12 developed apnoea 4–39 days after immunisation. 8 of these infants had other infective causes for their presentation, including late-onset GBS. **SAEs** included 6 infants admitted for apnoea requiring immediate endotracheal intubation and transfer to the intensive care unit for ongoing mechanical ventilation.
Immunogenicity and reactogenicity of DTPa-HBV-IPV/HiB vaccine as primary and booster vaccination in low-birth-weight premature infants. Vazquez et al., 2008 [23]	24–36 weeks	2, 4 and 6, 18–24 months	DTPa-HepB-IPV-Hib (Infanrix Hexa)	161	AEFIs included infants experiencing mild symptoms such as irritation or fever. The authors stated that the vaccine was well tolerated in preterm infants. No SAEs identified.
Frequency of respiratory deterioration after immunisation in preterm infants Hacking et al., 2010 [24]	Mean 27 weeks	2 months	DTPa-HiB Oral poliomyelitis (OPV), IPV Rotavirus	411	**SAEs** identified included 24 (5.8%) infants experiencing post-immunisation apnoea, requiring intermittent positive pressure ventilation or continuous positive airway pressure within 7 days of immunisation. These infants had a higher incidence of previous septicaemia or were more likely to have received CPAP for a longer period prior to vaccination. Of the 24 infants, 2 were diagnosed with septicaemia within 7 days of administration of the vaccine.
Very low birth weight infants have only few adverse events after timely immunization Furck et al., 2010 [25]	Median 28 weeks	2 months	Three different vaccine combinations: (A) DTP, IPV, Hib and Gen H-B-Vax K pro infantibus; (B) DTP, IPV, Hib, Hep B; (C) DTP, IPV, Hib, Hep B and pneumococcal serotype.	473	AEFIs included infants showing apnoea post-vaccination who were born, on average, 1.4 weeks earlier than those without apnoea. 51 (10.8%) infants presented with apnoea and/or bradycardia. There was an increased risk of bradycardia if there was apnoea. 13 (2.8%) presented with local reactions and/or fever. No SAEs identified.
Recurrent apnoea post immunisation: Informing re-immunisation policy Clifford et al., 2011 [26]	<37 weeks	2 and 4 months	DTPa-HepB-IPV-Hib (Infanrix hexa) or Haemophilus b Conjugate, Meningococcal Protein Conjugate and Hepatitis B (Recombinant) Vaccine (Infanrix-IPVTM and Comvax) and, 13-valent pneumococcal conjugate vaccine (PrevenarTM) and live attenuated pentavalent human–bovine reassortant rotavirus vaccine (RotateqTM)	38	Special cohort: 38 preterm infants who had experienced apnoea post-immunisation were studied in this paper. Further AEFIs included 35 of these infants who presented with apnoea again following the 2-month immunisation, whilst the other 3 infants did not at this time point but did during 4-month immunisations. One infant who developed apnoea following the 4-month immunisations had suffered a hypotonic hyporesponsive episode following their 2-month immunisations. **SAEs** included seven infants (18%) who had recurrent apnoea following immunisations. Even though these infants did not have any serious sequelae following this, recurrent apnoea qualifies as an SAE.
Apnoea after the 2-month immunisation in extremely preterm infants: What happens with the 4-month immunisation? Anderson et al., 2013 [27]	<29 weeks Mean birth gestation (babies with reactions) 26.0 ± 1.5 Mean birth gestation babies without reactions: 26/7 ± 1.2	2 and 4 months	DTPa-HepB-IPV-Hib and (Infanrix-hepB and Pedvax) DTPa-HepB-IPV-Hib and 13-valent pneumococcal conjugate vaccine (Infanrix-hepB, Pedvax and Prevenar) DTPa-HepB-IPV and 13-valent pneumococcal conjugate vaccine, (Infanrix-hexa and live attenuated pentavalent human–bovine reassortant rotavirus vaccine Prevenar and RotaTeq)	203	AEFI included a clinically significant apnoea occurred in 17/203 (8.4%) of the babies following their 2-month vaccinations. At the 4-month vaccination, 9 babies were vaccinated whilst having cardiorespiratory monitoring. 0/9 of these babies had AE. **SAEs** included 9/17 babies where a clinically significant apnoea occurred, required readmission to intensive care for respiratory support (CPAP or high-flow nasal cannula).
Respiratory Decompensation and Immunization of Preterm Infants Montague et al., 2016 [28]	<32 weeks	2 months	IPV, DTaP (Infanrix), Hib (Pedvax), hepatitis B vaccine (Engerix-B), pneumococcal conjugate vaccine (PCV13), influenza vaccine (Fluzone), rotavirus vaccine (Rotateq), and DTaP-IPV-HBV combination vaccine (Pediarix)	240	AEFIs were monitored for 72 h post immunisation. Of note, 172 (72%) had a diagnosis of BPD prior to administration of the vaccine. No statistically significant difference in respiratory decompensation, apnoea, bradycardia and desaturation events. No SAEs were identified.
Post-marketing surveillance study of the DTaP2-IPV-HB-Hib (Hexyon) vaccine administered in preterm infants in the Apulia region, Italy, in 2017 Martinelli et al., 2020 [29]	<37 weeks	3 months	DTaP2-IPV-HB-Hib (Hexyon) vaccine	700	AEFIs included 35.7% (n = 339) who reported local pain was the most common reaction with erythema, swelling, induration and nodule formation. No SAEs were identified.
Cardiorespiratory Events (CRE) Following the Second Routine Immunization in Preterm Infants: Risk Assessment and Monitoring Recommendations Bohnhorst et al., 2021 [30]	<31 weeks	3 months	DTPa-HBV-IPV/Hib (Infanrix hexa) and Pneumococcal polysaccharide conjugate vaccine (13-valent, adsorbed) (Prevenar 13)	71	Special cohort: This is a prospective observational study that included infants who required in-hospital monitoring because of increased recurrence of cardiorespiratory events during first immunisation. Further AEFIs included all but seven infants (90.1%) who showed an increase in cardiorespiratory events after the second routine immunisation. No infant required intermittent positive pressure ventilation or initiation of mechanical ventilatory support. No SAEs were identified.
Five year follow up of extremely low gestational age infants after timely or delayed administration of routine vaccinations Fortmann et al., 2021 [31]	<29 weeks	2 months	DTP-IPV-Hib-HepB (Hexavalent) pneumococcal vaccine: 7-, 10- or 13-valent conjugate vaccine.	8401	No AEFIs were reported. Timely immunised children (vaccination at 2 months after birth) had a lower risk of bronchitis (27.3% vs. 37.7%). No SAEs were identified.
Safety and immunogenicity of a fully-liquid DTaP-IPV-Hib-HepB vaccine (Vaxelis) in premature infants Wilck et al., 2021 [32]	<37 weeks	≥6 weeks	DTaP-IPV-Hib-HepB vaccine (Vaxelis)	160	The incidence of AEFIs such as injection site reactions was very high in term and preterm populations. The study reports **SAEs** occurred in term and preterm infants at 1.5% vs. 1.8% (difference not statistically significant). The SAEs are not further described. However, there were no cases of apnoea or cardiopulmonary events associated with vaccination.
Vaccination experiences of premature children in a retrospective hospital-based cohort in a Chinese metropolitan area Jin et al., 2021 [33]	≤37^+6^ weeks	≥1 months	BCG, DTaP, hepatitis A vaccine (HepA), HepB, Japanese encephalitis vaccine (JEV), measles–mumps–rubella vaccine (MMR), measles–rubella vaccine (MR), meningococcal serogroup A polysaccharide vaccine (MenA) and polio vaccine (PV)	1124	3.1% of infants had AEFIs following immunisation, which were mild in severity with one allergic rash reported.
Safety of Vaccination within First Year of Life - The Experience of One General Medicine Center Pop et al., 2023 [34]	<37 weeks and term controls	2, 4 and 11 months	Monovalent anti-hepatitis B vaccine, BCG vaccine and Hexavalent vaccine—against DTP-Hib-HepB-poliomyelitis Anti-pneumococcal vaccine (Prevenar 13) MMR Supplementary dose of MMR vaccine	81	There was no difference in the incidence and severity of AEFI within 12 h after immunisation between term and preterm infants. No SAEs were identified.

**Table 2:** Summary of safety outcomes of vaccines containing hepatitis B given to preterm infants within 7 days of birth. Abbreviations used: AEFI, adverse event following immunisation; SAE, serious adverse event.

Name of Study, Author	Gestation at Birth	Time Vaccination Was Given Post Birth	Vaccine Administered	n	Outcomes Summary, Including AEFIs Following Hepatitis B Immunisation
Hepatitis B vaccination in premature and low birth weight (LBW) babies Bhave, 2002 [35]	GA < 34 weeks (n = 25) GA 34 to 36 weeks (n = 25) Full term < 2.5 kg (LBW babies) (n = 25) Full term > 2.5 kg (n = 25)	0, 1, 2 and 12 months	Haemophilus b Conjugate (Comvax) (Meningococcal Protein Conjugate) and Hepatitis B (Recombinant) Vaccine), DTPa-HepB-IPV-Hib (Infanrix)	100	AEFIs were monitored after the immunisation, and up to 48 h following immunisation by the infants’ parents. The vaccine was well tolerated and safe. No SAEs were identified.
Vaccination recommendations, immunization status and safety of vaccination for premature infants in Zhejiang, China Xu, 2020 [36]	<37 weeks	Birth, 1 month, 6 months	HepB vaccine, BCG vaccine, IPV, Oral live attenuated polio vaccine, DTPa, Japanese encephalitis vaccine, Hepatitis a vaccine, Meningococcal meningitis-A vaccine, Meningococcal meningitis-AC vaccine, Measles, mumps, rubella vaccine and Diphtheria tetanus vaccine	1515	Seven cases experienced mild and self-limiting AEFIs including low-grade fever, local redness and swelling at injection site. No SAEs were identified.
Type 2 immune polarization is associated with cardiopulmonary disease in preterm infants Lao, 2022 [8]	24–29 weeks	Birth, 2 months	At birth: HepB (e.g., Engerix B) At 2 months: DTPa-HepB-IPV-Hib (Infanrix Hexa)	51	Compared to infants without BPD, infants with BPD exhibited ≤36-fold more Th2-polarised T cells. Early vaccination against hepatitis B was associated with increased Th2-polarisation. No SAEs were identified.

**Table 3:** Summary of AEFI rate seen in included studies. Abbreviations used: AEFI, adverse event following immunisation; SAE, serious adverse event.

	Number of Studies	Total Infants Studied	AEFI Rate (%), Comments
No AEs	1 study [35]	100	No AEFIs noted.
AEFIs in <10% of cases	7 studies [23,24,27,32,33,34,36]	3655	AEFIs such as low-grade fever and/or local reactions occurred in 0.4%. Transient episodes of apnoea occurred in 0–5.8%.
AEFIs in >10% of cases	9 studies [17,18,19,20,21,22,25,29,32]	1833	AEFIs such as low-grade fever and/or local reactions occurred in 14–96%. Transient episodes of apnoea and/or bradycardia occurred in 10–13%
Any SAEs	6 studies [20,22,24,26,27,32]	1051	AEFIs included 11% of infants who developed cardiorespiratory events that required oxygen therapy or an increase in FiO_2_, whilst 5% were commenced on bag-mask ventilation, CPAP, mechanical ventilation or an increase in ventilation settings. 26% of infants admitted for apnoea required immediate endotracheal intubation and transfer to the intensive care unit for ongoing mechanical ventilation. 5.8% of infants with post-immunisation apnoea required intermittent positive pressure ventilation or CPAP.
Included term vs. preterm	3 studies [19,32,34]	333	12.0% vs. 14.2% for fever in term vs. preterm. 1.5% vs. 1.8% in SAEs in term vs. preterm. Differences between term and preterm infants were not statistically significant.
Included single-antigen hep B vaccine	3 studies [8,35,36]	1666	Our own study was not designed to assess safety of the hepatitis B vaccine [8]. The other two studies did not specifically report on AEFIs of the birth dose.

## Data Availability

Data is contained within the article (and Supplementary Materials).

## Supplementary Materials

The following supporting information can be downloaded at: https://www.mdpi.com/article/10.3390/vaccines12030261/s1, Figure S1: Risk of bias assessment of included clinical studies using. Abbreviations used: +, low risk of bias; ?, unclear risk of bias; −, moderate risk of bias; Table S1: Search strategy applied to (A) MEDLINE via Ovid 1946 to 30 March 2023; (B) Embase via Ovid 1947 to 30 March 2023; (C) Cochrane Central Register of Controlled Trials via 30 March 2023; (D) CINAHL Plus 30 March 2023.
