# Supplementary material for: Safety of Hepatitis B Vaccines (Monovalent or as Part of Combination) in Preterm Infants: A Systematic Review
Source: Vaccines (Basel). 2024 Mar 1;12(3):261. doi: 10.3390/vaccines12030261 (PMC10974192; doi:10.3390/vaccines12030261)
Supplement: Supplementary file 1 [file vaccines-12-00261-s001.zip › vaccines-2849430-supplementary.pdf]

Table S1. *Search strategy applied to (A) MEDLINE via Ovid 1946 to March 30, 2023; (B) Embase via Ovid 1947 to March 30, 2023; (C) Cochrane Central Register of Controlled Trials via March 30, 2023 (D) CINAHL Plus March 30, 2023.*

(A)

#	Query
1	Patient Safety/ or Safety/ or safety.mp.
2	Population Surveillance/ or surveillance.mp.
3	Complication.mp.
4	Adverse event.mp.
5	Response.mp.
6	Deterioration.mp. or Clinical Deterioration/
7	1 or 2 or 3 or 4 or 5 or 6
8	Viral Vaccines/ or vaccin*.mp. or Vaccination/ or Vaccines/
9	Immunis*.mp.
10	Immunization/ or immuniz*.mp. or Immunization Programs/
11	Inoculat*.mp.
12	Infant, Premature/ or Premature Birth/ or preterm.mp.
13	Prematur*.mp.
14	8 or 9 or 10 or 11
15	12 or 13
16	Infant.mp. or Infant, Newborn/ or Infant/ or Infant, Premature, Diseases/ or Infant, Extremely Premature/

17	Neonat*.mp. or Neonatology
18	Baby.mp.
19	Babies.mp.
20	16 or 17 or 18 or 19
21	7 and 14 and 15 and 20
22	Limit 21 to last 20 years

(B)

#	Query
1	Patient Safety/ or Safety/ or safety.mp.
2	Surveillance.mp. or monitoring
3	Complication/ or complication.mp.
4	Controlled study/ or adverse event.mp. or adverse event/ or clinical trial/
5	Response.mp. or cardiovascular response/
6	Deterioration.mp. or deterioration/
7	1 or 2 or 3 or 4 or 5 or 6
8	Vaccination/ or vaccin*.mp. or vaccine/
9	Virus vaccine/ or immunis*.mp.
10	Immunization/ or immuniz*.mp.

11	Inoculation/ or inoculat*.mp.
12	8 or 9 or 10 or 11
13	Prematurity/ or preterm.mp.
14	Prematur*.mp.
15	13 or 14
16	Infant disease/ or high risk infant/ or infant.mp. or hospitalized infant/
17	Neonatology/ or newborn/ or neonatal intensive care unit/ or neonat*.mp.
18	Baby.mp. or baby/
19	Babies.mp.
20	16 or 17 or 18 or 19
21	7 and 12 and 15 and 20
22	Limit 21 to last 20 years

(C)

#	Query
1	Patient Safety/ or Safety/ or safety.mp.
2	Surveillance.mp.
3	Complication.mp.
4	Adverse event.mp.
5	Generalization, response/ or response.mp.

6	Deterioration.mp. or Clinical Deterioration/
7	1 or 2 or 3 or 4 or 5 or 6
8	Vaccin*.mp.
9	Immunis*.mp.
10	Immunization/ or immuniz*.mp.
11	Inoculat*.mp.
12	8 or 9 or 10 or 11
13	Preterm.mp. or Infant, Premature/
14	Premature Birth/ or prematur*.mp.
15	13 or 14
16	Infant, Extremely Premature/ or infant Health/ or infant.mp. or Infant/ or Infant, Newborn/ or Infant, Newborn, Diseases/ or Infant, Premature, Diseases/
17	Neonat*.mp.
18	Baby.mp.
19	Babies.mp.
20	16 or 17 or 18 or 19
21	7 and 12 and 15 and 20
22	Limit 21 to last 20 years

(D)

#	Query
1	Safety
2	Surveillance
3	Complication
4	Adverse event
5	Response
6	Deterioration
7	S1 or S2 or S3 or S4 or S5 or S6
8	Vaccin*
9	Immunis*
10	Immuniz*
11	Inoculat*
12	S8 or S9 or S10 or S11
13	Preterm
14	Prematur*
15	13 or 14
16	Infant
17	Neonat*

18	Newborn
19	Baby
20	Babies
21	S16 or S17 or S18 or S19 or S20
22	S7 and S12 and S15 and S21
23	Limit S22 by Publish date (2003/03/01 to 2023/03/31)

Figure S1. *Risk of bias assessment of included clinical studies using. Abbreviations used: +, low risk of bias; ?, unclear risk of bias; -, moderate risk of bias.*

	Bias due to confounding	Bias in selection of participants into the study	Bias in classification of interventions	Bias due to deviation from intendend interventions	Bias due to missing data	Bias in measurement of outocmes	Bias in selection of the reported results
Anderson et al 2013	+	-	?	+	?	-	+
Bhave et al 2002	+	+	?	+	-	+	+
Bohnhorst et al 2021	+	+	+	+	+	-	?
Clifford et al 2011	+	+	+	+	+	-	+
Cooper et al 2008	+	+	+	+	?	+	+
Ellison et al 2005	+	?	?	+	?	+	+
Faldella et al 2007	?	?	+	?	?	-	+
Fortmann et al 2021	+	+	+	?	+	-	+
Furck et al 2010	?	+	+	+	?	-	+
Hacking et al 2010	?	?	?	+	+	-	+
Jin et al 2021	?	?	?	?	?	-	+
Lao et al 2022	?	+	+	?	+	-	?
Martinelle et al 2020	+	?	?	?	?	-	?
Montage et al 2016	?	-	?	?	+	-	+
Omenaca et al 2005	?	+	+	+	?	-	+
Pop et al 2023	?	?	?	+	?	-	?
Pourcyrous et al 2007	+	+	+	+	+	-	+
Schulzke et al 2005	?	+	+	+	?	-	+
Vazquez et al 2008	?	+	+	+	?	-	+
Wilck et al 2021	+	+	+	+	+	+	+
Xu et al 2020	?	?	?	+	?	-	?
